# Supplementary material for: Breunnerite grain and magnesium isotope chemistry reveal cation partitioning during aqueous alteration of asteroid Ryugu
Source: Nat Commun. 2024 Sep 5;15:6809. doi: 10.1038/s41467-024-50814-y (PMC11377773; doi:10.1038/s41467-024-50814-y)
Supplement: Supplementary file 1 — Supplementary Information [file 41467_2024_50814_MOESM1_ESM.pdf]

Supplementary Information for

"Breunnerite grain and magnesium isotope chemistry reveal cation partitioning during aqueous alteration of asteroid Ryugu"

Toshihiro Yoshimura[1*], Daisuke Araoka[2], Hiroshi Naraoka[3], Saburo Sakai[1], Nanako O. Ogawa[1], Hisayoshi Yurimoto[4], Mayu Morita[5], Morihiko Onose[5], Tetsuya Yokoyama[6], Martin Bizzarro[7], Satoru Tanaka[5], Naohiko Ohkouchi[1], Toshiki Koga[1], Jason P. Dworkin[8], Tomoki Nakamura[9], Takaaki Noguchi[10], Ryuji Okazaki[3], Hikaru Yabuta[11], Kanako Sakamoto[12], Toru Yada[12], Masahiro Nishimura[12], Aiko Nakato[12], Akiko Miyazaki[12], Kasumi Yogata[12], Masanao Abe[12], Tatsuaki Okada[12], Tomohiro Usui[12], Makoto Yoshikawa[12], Takanao Saiki[12], Satoshi Tanaka[12], Fuyuto Terui[13], Satoru Nakazawa[12], Sei-ichiro Watanabe[14], Yuichi Tsuda[12], Shogo Tachibana[12,15] and Yoshinori Takano[1*]

---

[1] Biogeochemistry Research Center (BGC), Japan Agency for Marine-Earth Science and Technology (JAMSTEC), Natsushima 2-15, Yokosuka, Kanagawa 237-0061, Japan.

[2] Geological Survey of Japan (GSJ), National Institute of Advanced Industrial Science and Technology (AIST), 1-1-1 Higashi, Tsukuba, Ibaraki 305-8567, Japan.

[3] Department of Earth and Planetary Sciences, Kyushu University, 744 Motooka, Nishi, Fukuoka 819-0395, Japan.

[4] Creative Research Institution (CRIS), Hokkaido University, Sapporo, Hokkaido 001-0021, Japan.

[5] HORIBA Techno Service Co., Ltd., Kisshoin, Minami-ku Kyoto 601-8510, Japan.

[6] Department of Earth and Planetary Sciences, Tokyo Institute of Technology, Ookayama, Meguro, Tokyo 152-8551, Japan.

[7] Centre for Star and Planet Formation, Globe Institute, University of Copenhagen, Copenhagen, K 1350, Denmark.

[8] Solar System Exploration Division, NASA Goddard Space Flight Center, Greenbelt, Maryland 20771, U.S.A.

[9] Department of Earth Science, Tohoku University, Sendai 980-8678, Japan.

[10] Department of Earth and Planetary Sciences, Kyoto University, Kyoto 606-8502, Japan.

[11] Earth and Planetary Systems Science Program, Hiroshima University, Higashi Hiroshima 739-8526, Japan.

[12] Institute of Space and Astro-nautical Science, Japan Aerospace Exploration Agency (ISAS/JAXA), Sagamihara, Kanagawa 229-8510, Japan.

[13] Kanagawa Institute of Technology, Atsugi 243-0292, Japan

[14] Department of Earth and Planetary Sciences, Nagoya University, Nagoya 464-8601, Japan.

[15] UTokyo Organization for Planetary and Space Science (UTOPS), University of Tokyo, Bunkyo, Tokyo 113-0033, Japan.

* equal contribution.

Corresponding authors:

e-mail: yoshimurat@jamstec.go.jp, takano@jamstec.go.jp

Contents

- List of Abbreviations.
- Percentages of dolomite-derived carbon to total carbon in samples A0106 and C0107.
- Mass-dependent Mg isotope fractionation and precipitation temperature of dolomite.
- Mg isotope fractionation of clay minerals.
- Assessing the effect of radiogenic ^{26}Al decay on δ^{26}Mg*.
- The role of sodium in interactions with soluble organic matter and minerals.
- Supplementary Figures S1–S6.
- Supplementary Tables S1–S5.
- Supplementary References.

List of Abbreviations

C-type	Carbonaceous-type
CI-type	Ivuna type
CM-type	Mighei-type
ICP-MS	Inductively coupled plasma mass spectrometry
XRF	X-ray fluorescence spectroscopy
EDTA	Ethylenediaminetetraacetic acid
SOM	Soluble organic matter
SCI	Small Carry-on Impactor.

Supplementary text

Percentages of dolomite-derived carbon to total carbon in samples A0106 and C0107

The total carbon content of A0106 was reported by Naraoka et al. (2023) to be 3.76 wt%[1], and that of C0107 was reported by Oba et al. (2023) to be 3.58 wt%[2] (Supplementary Figure S4A). The content of dolomite, the most abundant carbonate mineral of Ryugu, was calculated from the amount of calcium in the formic acid (HCOOH) extract (the carbonate fraction of the sequential solvent extraction method) to be 91.3 μmol/g in A0106, and 112.9 μmol/g in C0107[3]; the Mg/Ca atomic ratio of dolomite (chamber A = 0.98 ± 0.04, n = 52; chamber C = 0.98 ± 0.04, n = 49)[4] was used in this calculation. The inorganic carbon content varied considerably from sample to sample (see below). Therefore, the dolomitic C abundance relative to total C specific to our samples was calculated to be 5.77 ± 1.46% (2SD) for A0106 and 7.49 ± 1.14% for C0107.

Supplementary Figure S4B shows how the carbonate contents vary in relation to the sum of phyllosilicates and carbonates. The percentage of carbonate was converted to wt% by using the mineral densities from Dartois et al. (2023)[5]. The infrared spectroscopy was generally obtained for a field of view of approximately 30 to 400 μm in size, but it included carbonate-enriched sites at 2 of 18 measurement points in chamber A and at 2 of 14 measurement points in chamber C. A similarly high carbonate content was also reported by Moynier et al. (2022) with a 2-fold difference in concentration

between bulk A and C samples[6], and by Ito et al. (2022) with a variation of 2–21 vol% in a micro-area analysis[7]. Yokoyama et al. (2023) reported that 90% of the total carbon content of 3.31 ± 0.33 wt% was derived from organic carbon in the case of Ivuna, but in the case of Ryugu, 3.08 ± 0.30 wt% of the total carbon content of 4.63 ± 0.33 wt% was organic carbon[8]. These results indicate large inter-grain variation in non-organic carbon contents.

Mass-dependent Mg isotope fractionation and precipitation temperature of dolomite

Schauble (2011) and Rustad et al. (2010) estimated the degree of Mg isotope fractionation ($\Delta^{25}Mg$) between dissolved Mg and carbonates by using a density function[9, 10] and assuming the Mg^{2+} ions to be an infinitely diluted species in water (according to Li et al., 2015), which may not be an adequate approximation of Ryugu precipitation conditions. The isotope fractionation factors for dolomite obtained from these studies deviate both positively and negatively from the fluids.

Carbonates obtained under natural conditions or by inorganic precipitation are commonly enriched in ^{24}Mg relative to the solution. Li et al. (2015) precipitated dolomite from a solution of 1M $MgCl_2$ and 1M $CaCl_2$ and reported the fractionation equation[11]. This fractionation equation is in good agreement with the $\Delta^{25}Mg_{dolomite\text{-}aq.}$ value of −1.0‰ to −1.35‰ obtained from dolomite precipitated from pore water in oceanic sediments which is considered to be close to equilibrium isotope fractionation because the precipitation reaction is very slow[12, 13]. Fractionation factors were also obtained from sedimentary[12] and Sabkha[14] dolomite and porewater, but the $\Delta^{25}Mg_{dolomite\text{-}aq.}$ values from the precipitation experiments of Li et al. (2015)[11] were chosen because the influence of porewater migration and impurities cannot be ruled out[e.g., 14]. To show the influence of different fractionation factors, the calculation results using the fractionation factor proposed by Fantle and Higgins et al. (2014)[12] are also shown in Supplementary Figures S6B, S6D, and S6F. The reaction temperature of these sediments ranges from 4.5 to 17.4 °C. In this study, the $\Delta^{25}Mg$ value of dolomite was calculated using the fractionation equation of Li et al. (2015)[11] based on the similarity of the precipitation conditions and the consistency with natural dolomite. The dolomite precipitation temperature of Ryugu was 37 ± 10 °C[8]. Based on their composition and lattice constants, the formation and equilibrium temperature of the secondary mineral pentlandite is 25 °C and that of the

secondary mineral pyrrhotite is 20 ± 29.5 °C, and the low abundance of Mg chlorite is also consistent with low-temperature conditions[15]. The estimated temperatures of aqueous alteration are all in good agreement.

The isotopic composition of Mg^{2+} supplied by the reaction of water with primary minerals in the Ryugu parent body is the same value as that of C0108[16]. The bulk values of the small samples from Ryugu are affected by carbonates, but the values of these samples are indistinguishable from previously reported values for CI meteorites[16]. Based on the following four constraints, the contribution of dolomitic Mg to the total Mg content in Ryugu was estimated: (i) the carbon content of dolomite shown in Supplement Figure S4; (ii) the total carbon content of A0106 and C0107[1, 17]; (iii) the molecular weight of dolomite calculated from the Mg/Ca ratio in Supplement Table S2; and (iv) the total Mg concentration of A0106 and C0107 samples[8]. The degree of dolomitic Mg present is estimated to be 2.0% for A0106 and 2.4% for C0107. This is close to the model conditions of Supplementary Figures S6A and S6B. In addition, the amount of exchangeable ions in phyllosilicates relative to dolomitic Mg is calculated, using the following three constraints: (I) the percentage of exchangeable Mg in Supplementary Table S5; (II) the extracted Mg and Ca in the chemical extracts[3]; (III) Ca in Ryugu is mostly derived from carbonate minerals[6]. Exchangeable Mg was calculated as 47% of dolomitic Mg. From these conditions, the three relative partitioning amounts of phyllosilicate, dolomite, and exchangeable Mg in aqueous alteration were calculated, and the results are shown in Supplementary Figure S6 for the three Mg partitioning ratios in the model calculation. All conditions support the precipitation of dolomite late in the degree of Mg loss associated with aqueous alteration. The results for the conditions where the fractionation coefficients for clay minerals are close to the reported values and the abundance of dolomitic Mg is close to the above calculated values (Supplementary Figures S6A and S6C) are shown in Figures 7A and 7B as representative results in this partitioning model.

Mg isotope fractionation of clay minerals

The Ryugu samples are mixtures of water-reactive inorganic minerals and organic matter with very high porosity. The hot H_2O extract from the SOM leaching contained anions desorbed from the organic material[3]. The contribution of Mg from the salt and organic phases was small. The factor controlling the chemical composition of the H_2O and NH_4Cl extracts was Mg from soluble and exchangeable components in

phyllosilicates (especially clay minerals), which are the main lithological constituents of Ryugu as well as the dominant host phase for Mg[cf. 18]. Hashiguchi et al. (2023), who conducted imaging mass spectrometry, reported that the organic molecular groups and lithological Mg-Fe elements are spatially heterogeneous on a sub-millimeter scale (~100 μm)[18].

With regard to the isotopic fractionation of Mg between the aqueous phase and minerals (Δ^{25}Mg and Δ^{26}Mg) during its structural incorporation into phyllosilicates, it has been shown that ^{25}Mg and ^{26}Mg are preferentially incorporated into the mineral phase during natural weathering, as shown by paired analyses of rivers and rocks and bulk clay minerals[19, 20]. An isotopic fractionation factor of α (^{26}Mg/^{24}Mg) = 1.0005 has been shown experimentally for brucite, a type of layered mineral[21]. Although α is specific to each phyllosilicate mineral[22], the α value for ^{26}Mg/^{24}Mg fractionation of secondary phyllosilicates calculated from the mass balance model for aqueous alteration of Ryugu (Supplementary Figure S6) is 1.00053 (1.00027 for ^{25}Mg/^{24}Mg) for the condition of Supplementary Figure 6C, which is consistent with the value reported by Wimpenny et al. (2014)[21].

Assessing the effect of radiogenic ^{26}Al decay on δ^{26}Mg*

The age of the aqueous alteration of carbonaceous chondrites has been constrained by the ^{53}Mn–^{53}Cr ages of carbonates[23-25], and although aqueous alteration histories differ among CI, CM, and Tagish Lake meteorites, many carbonaceous chondrite parent bodies accreted almost simultaneously around 4.4–5.7 million years (Myr) after calcium–aluminum-rich inclusion (CAI) formation[24]. The heat source responsible for the aqueous alteration is proposed to derive from the radiogenic decay of short-lived nuclides, mainly ^{26}Al. Dolomite and magnetite precipitation at Ryugu occurred at 5.2 Myr (+0.8 Myr, –0.7 Myr), after CAI formation (calculated from initial Mn/Cr values for the D'Orbigny meteorite)[8]. Carbonate has been reported to be one of the earliest minerals to precipitate during alteration processes of other Ryugu grains, and two studies[26, 27] have indicated an older age for its precipitation, that is, within 1.8 Myr after CAI formation[26]. Mn-Cr ages of breunnerite and dolomite of CI chondrites show that breunnerite precipitates later than dolomite[e.g., 25]. Although it has been pointed out that measurement biases due to matrix differences in secondary ion mass spectrometry analyses may be included, ^{26}Al decay-driven aqueous alteration of the CI parent body may have continued

to 4554.6 Myr ago. This age corresponds to that of the latest breunnerite for a parent body radius of >50 km, suggesting that the temperature was kept above the melting point of ice for >9 Myr from the time of dolomite precipitation (4563.6 Myr ago)[24]. As is apparent from the above ages, the secondary mineral precipitation events occurred late such that an excess of ^{26}Mg due to in situ decay of ^{26}Al is unlikely[e.g., 28]. The ^{27}Al/^{24}Mg ratio of carbonates, especially from Mg-bearing carbonates would be low, further supporting a low ^{26}Mg-excess if any.

The δ^{26}Mg* reflects the mass-independent fractionation related to the decay of ^{26}Al[29, 30]. The mass-independent isotopic fractionation of δ^{25}Mg and δ^{26}Mg is described by a power function with exponent β.

In this study, a β value of 0.511 was adopted to calculate δ^{26}Mg* values:

$$\delta^{26}\mathrm{Mg}^{*} = \{(1+\delta^{26}\mathrm{Mg}/1000) - (1+\delta^{25}\mathrm{Mg}/1000)^{1/\beta}\} \times 1000 \qquad \text{[Eq. S1]}$$

The δ^{26}Mg* value is usually applied to materials or minerals with high ^{27}Al/^{24}Mg such as chondrules, plagioclase (i.e., anorthite–albite; $CaAl_2Si_2O_8$, $NaAlSi_3O_8$), and glass[e.g., 29]. Recently, high-precision Mg isotope analysis has made it possible to evaluate mixing in common elemental reservoirs of, for example, CAIs and other silicates, and apparently ^{26}Al and ^{26}Mg are not correlated in bulk samples of various chondrites[31]. Although in recent years, ultra-precise analyses at the level of a few ppm have been carried with microgram-scale amounts of Mg[28, 32-34], the accuracy of the isotope ratio measurements in this study, which used tens to hundreds of nanograms of Mg extract, was generally around 50 ppm.

Assessing partial dissolution of phyllosilicates and carbonates during the sequential leaching experiment

Neutral solutions of NH_4Cl should not cause breakdown of the clay structure[21], but the Ryugu sample is highly reactive and the mineral lattice may be slightly broken by reaction with the solution. The exchangeable pools of phyllosilicate minerals consist of Mg adsorbed on interlayer and surface sites by electrostatic interactions. These adsorbed cations are relatively weakly bound and can be easily exchanged by using NH_4Cl. In contrast, in the smectite family, which includes saponite, a typical Ryugu secondary silicate mineral, the majority of Mg is structurally bound due to isomorphous exchange

of Mg^{2+} and Al^{3+} in the octahedral layer. Therefore, as the structural Mg contamination could be due to the dissolution of the phyllosilicates themselves, we tried to estimate the influence of the structural component of the saponite from the Al concentration in our extraction solutions.

The Mg/Al ratios of the solutions are shown in Table 2. The highest Mg to Al volume ratio is found in the NH_4Cl extract, where Mg is 36 times higher in A0106 and about 650 times higher in C0107. This may reflect the fact that the NH_4Cl solution successfully extracts the exchangeable ions without causing significant dissolution of the clay. Even assuming for simplicity that the structural Mg substituted with Al in a 3:2 ratio to keep the layer charge neutral is dissolved by congruent dissolution, the associated release of structural Mg is a few percent of the total Mg extracted. Furthermore, other cations are also present at the substitution site. The cation structurally replacing the Al^{3+} is most likely not only Mg, but also other cations such as Na. The main cation composition of the solution during actual aqueous alteration is shown in Supplementary Table S1, where Mg only accounts for 12.6% to 21.9% of the exchangeable fraction. Thus, the substitutable Mg would only have a low contribution to the exchangeable pool of Mg. Indeed, the Mg isotope ratios ($\delta^{25}Mg$) of these samples agree well at -0.67 ± 0.09‰ and -0.61 ± 0.04‰, respectively. The good agreement of the isotope ratios despite the large Mg/Al difference between the two samples supports the small influence of structural Mg.

On the other hand, the first extract, which represents the ultrapure water fraction, gives lower Mg/Al than the NH_4Cl extraction, namely 4.58 times Mg to Al in A0106 and 16.52 times Mg to Al in C0107. The effect of silicate dissolution is about an order of magnitude greater in these samples than in the NH_4Cl fraction. The $\delta^{25}Mg$ of C0107 is -0.68 ± 0.09‰, in good agreement with the NH_4Cl extract, while A0106 has a slightly lower value of -0.85 ± 0.02‰. The possibility of a larger contribution of silicate to A0106 can be ruled out, since $\delta^{25}Mg$ should be higher if secondary silicate dissolution is affected. In contrast, dolomite has a low value of -1.35‰[16, this study], which suggests that it may have dissolved slightly. In this case, the Mg/Ca ratio should approach 0.948 mol/mol, but the Mg/Ca of the ultrapure water fraction is 9.66 and no such trend is observed. This slightly lower $\delta^{25}Mg$ may therefore be due to slight isotopic heterogeneities in the clay mineral structure itself, with surface-bound Mg reported to have a predominantly lower isotopic ratio than the bulk and to be preferentially released by weak acids[21]. Moreover, extraction with hot H_2O also selectively releases components with similar isotopic compositions,

which may contribute to highly soluble salt materials[3]. Thus, there may be isotopic differences between surface-bound and interlayer cations, but in any case the effect of secondary silicate heterogeneity on the -0.71‰ isotopic composition of the exchangeable pool we determined (Supplementary Table S4) is minor, and the effect on the Mg distribution shown in Fig. 7 is limited compared to the carbonate fractionation factors discussed below.

The δ^{25}Mg compositions of the Ryugu carbonate leaching solutions, that is, the EDTA and CH_3COOH extracts, can be explained by the mixing of carbonate with components extracted by $HF/HClO_4$ (Fig. 4); the latter consist mainly of phyllosilicates and includes the most abundant component of Ryugu. The Ryugu carbonate leaching regents are selected to selectively extract carbonates, but some phyllosilicates, especially saponite, are dissolved into the leachate (Supplementary Table S1). In general, EDTA solutions are less reactive with clay minerals and thus only dissolve carbonates, but Ryugu's phyllosilicate minerals appear to be more reactive than terrestrial phyllosilicates and dissolve more easily into leachates.

The role of sodium in interactions with soluble organic matter and minerals

To understand the preservation of SOM in carbonaceous chondrites, knowledge of the processes at the organic–mineral interface is needed. Secondary phyllosilicate phases, such as saponite, which are characterized by a highly reactive surface, induce adsorption reactions. Negative charge sites on clay mineral surfaces not only act as adsorption sites for dissolved cations but also hold ionic SOMs by relatively weak electrostatic forces (Figure 8). Soluble ions that form direct chemical bonds are called adsorbed and interact with monodentate hydroxyl groups via ligand exchange, whereas non-ionic and non-polar molecules are essentially repelled from polar solutions and distributed to the non-polar molecular framework of organic matter[35]. Soluble organic matter with amphiphilic properties has also been detected on Ryugu[3], and such heterogeneous mixtures of SOMs are expected to be arranged in discrete zones on reactive mineral surfaces, where exposed hydrophobic parts of directly adsorbed amphiphilic molecules that form hydrophobic sites on other amphiphilic molecules may also be shielded from the polar aqueous phase[36].

With regards to the interaction of the main component cations with organic matter, univalent cations such as Na^+ and K^+ tend to act as bulk electrolytes that stabilize

surface charges, whereas multivalent cations such as Mg^{2+} bind with higher affinity and often play a specific structural or catalytic role[37]. Of the cation compositions identified in this study, multivalent cations, represented by Mg^{2+}, potentially form cationic cross-linking, but Hashiguchi et al. (2023) found no evidence of large amounts of organo-Mg compounds by *in situ* analysis of organometallic compounds on Ryugu[18]. However, organo-Na compounds are the most abundant type of metallo-organics in Ryugu, accounting for 4.6% of the signal intensity in desorption electrospray ionization–high-resolution mass spectrometry of the A0080 grain[18]. Sodium is known as a typical cation leading to the salting-out effect in the Hofmeister series (Figure 8), and charged or polar functional groups, such as carboxyl groups and sulfonic groups in organic materials, have been detected on Ryugu[1-3, 38]. The present study showed that Na^{+} was the most abundant dissolved cation during the aqueous alteration and that it played a role in stabilizing the surface charge of both inorganic and organic materials (i.e., secondary phyllosilicates and polar functional groups on organic materials).

Supplementary Figures

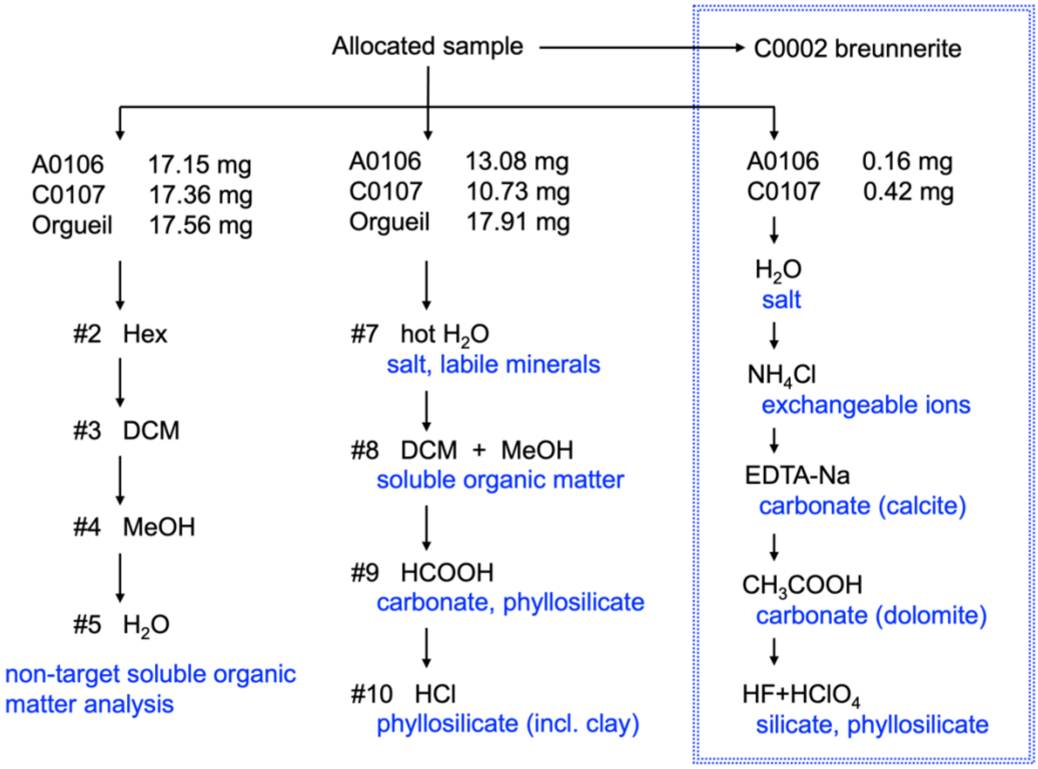

Supplementary Figure S1. Chart of sequential leaching experiments performed on initial samples A0106 and C0107 of asteroid (162173) Ryugu collected during the first and second touchdown sampling, respectively[39, 40], respectively. The sequential leaching performed in this study and the analyzed fractions are shown in the blue box on the right. Details of sample quality control and environmental assessment have been previously reported[40, 41]. The soluble organic matter (SOM) team performed the initial chemical leaching experiments of Ryugu samples[1, 17]: #2 to #5 (left) mainly used organic solvents to analyze hydrophobic SOM, and #7 to #10 (middle) focused mainly on hydrophilic SOM, co-working with chemistry team[8] (Yokoyama et al., 2023) and insoluble organic matter (IOM) team[42].

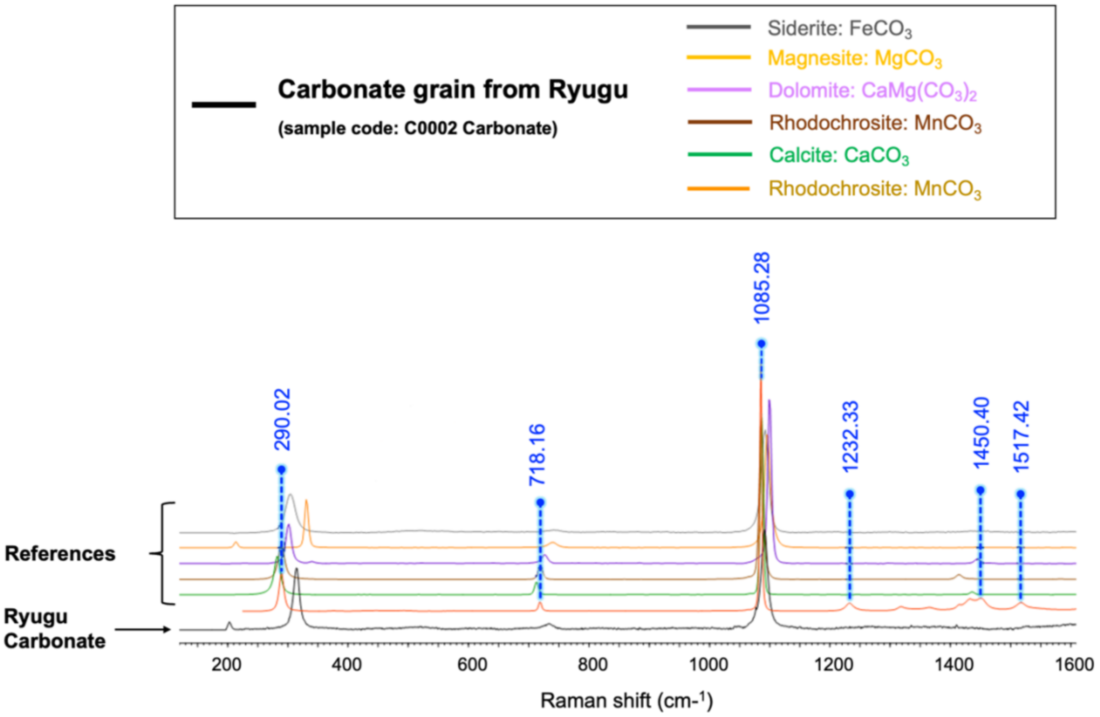

Supplementary Figure S2. Laser Raman Micro-Spectroscopy of a single carbonate grain from Ryugu C0002 and reference standards. The profiles of siderite ($FeCO_3$), magnesite ($MgCO_3$), dolomite ($CaMg(CO_3)_2$), rhodochrosite ($MnCO_3$), and calcite ($CaCO_3$) are shown. The analysis was conducted with a LabRAM HR Evolution Raman microscope (Horiba Ltd., Japan) using the method of Urashima et al. (2022)[43]. The orange spectrum is for Caltech-GRR#1858 reference code rhodochrosite ($MnCO_3$)[cf. 44]

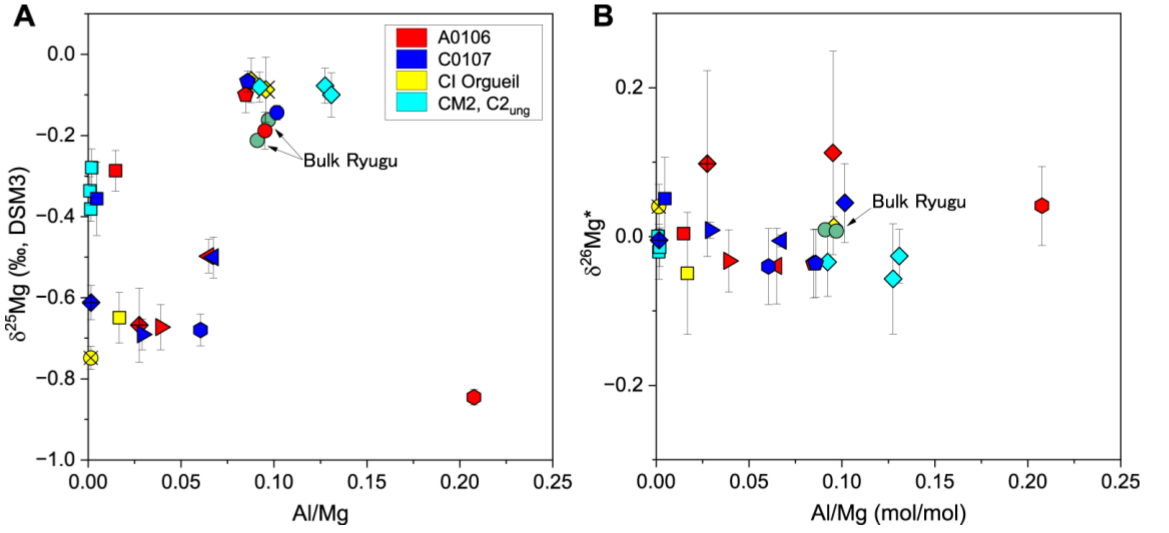

Supplementary Figure S3. Normalized magnesium isotope systematics of δ^{25}Mg and δ^{26}Mg for assessing potential ^{26}Al/Mg profiles at Ryugu.

(A) Mg isotopic ratios (δ^{25}Mg with 2SD errors) and **(B)** ^{26}Mg excess (δ^{26}Mg*) versus Al/Mg in the leaching fractions of this study and the SOM leaching fractions. See Figures 3 and 4 for the correspondence between the symbol shape and the leaching fraction. The secondary minerals, especially carbonates, have low initial ^{27}Al/^{24}Mg, and their precipitation age is about seven half-lives after CAI formation. After approximately 3 Ma (4 half-lives), δ^{26}Mg* cannot be distinguished from the Al/Mg partition model within the error range of 2 ppm[e.g., 28]. For further confirmation, we compared δ^{26}Mg* and Al/Mg to evaluate the possibility of elemental redistribution associated with alteration of the primary minerals. The blue-green circles show data from the bulk analysis of Ryugu grains[16].

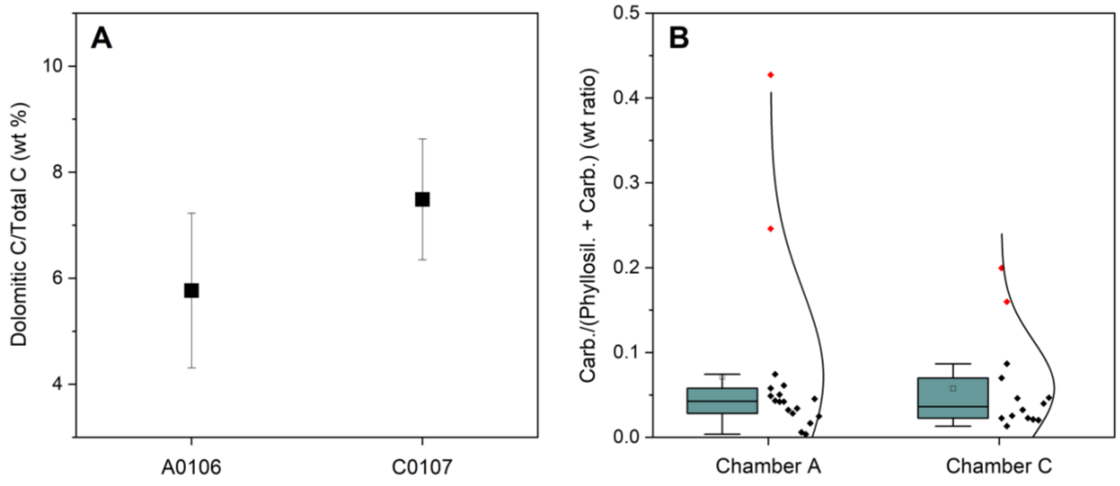

Supplementary Figure S4. Quantitative estimation of carbonate in the Ryugu sample. (A) Percentage of dolomite-derived carbon to total carbon content in samples A0106 (5.77 ± 1.46%, 2SD) and C0107 (7.49 ± 1.14%, 2SD), used for the dissolved organic matter analysis of Ryugu samples. **(B)** Box and whisker plots of the carbonate contents in relation to the sum of phyllosilicates and carbonates in A0064, C0002, C0040, C0046, C0057, A0106-13, A0108-15, A0108-19, C0109-4, and C0109-10 (black symbols) reported by Dartois et al. (2023)[5]. The black line indicates a lognormal distribution and red symbols are statistical outliers.

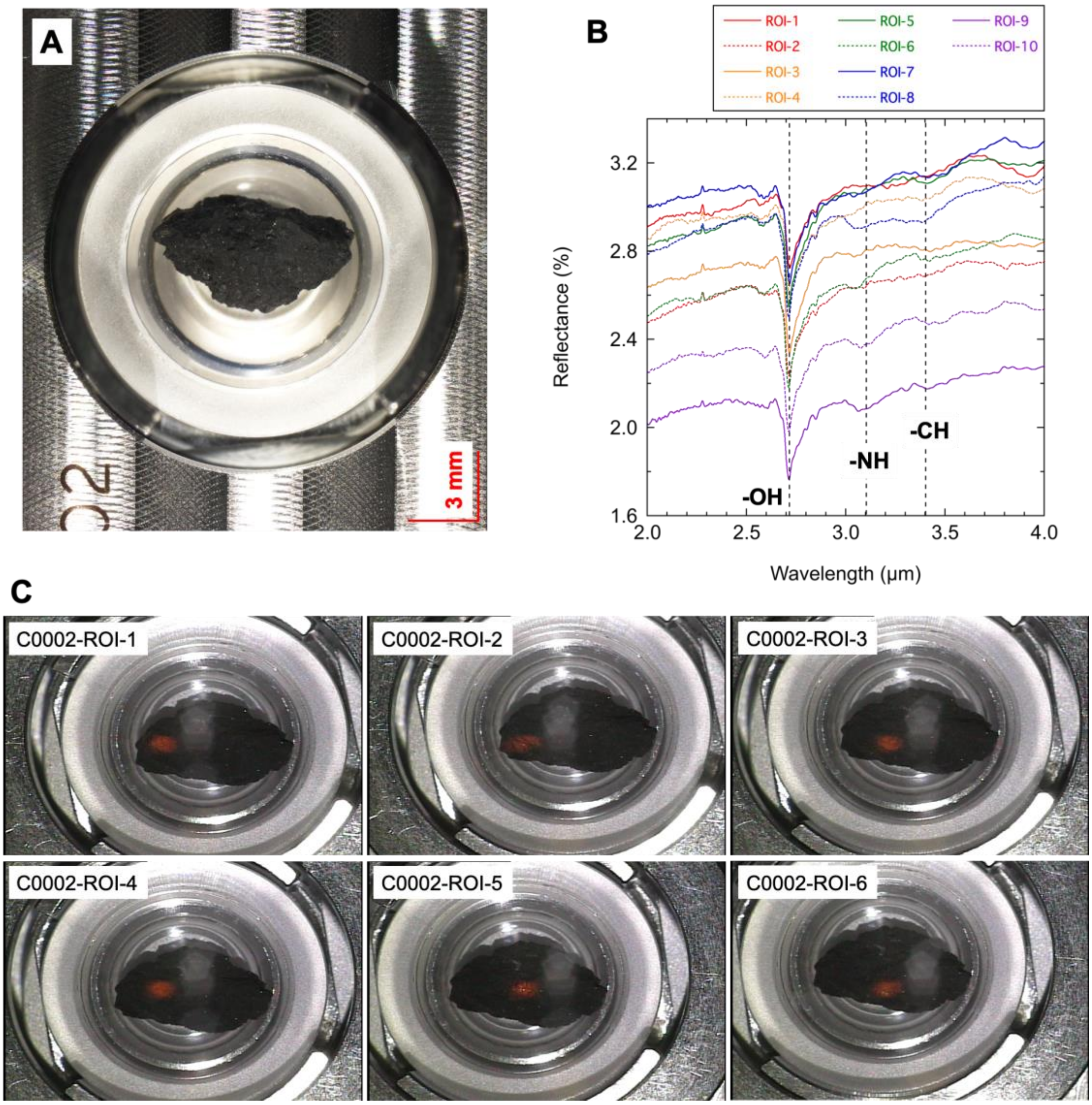

Supplementary Figure S5. Initial properties and spectral characteristics of Ryugu sample C0002. (A) Photograph of Ryugu C0002, **(B)** raw profiles of infrared reflectance spectra of the initial bulk sample (C0002) at wavelengths from 2.0 μm to 4.0 μm for each region of interest (ROI), and **(C)** photographs showing the positions of ROIs 1–6 of C0002. Data acquisition by this non-destructive analysis method is described in Yada et al. (2022)[45]. Signals for important functional groups (-OH, -NH, -CH) are indicated by vertical dotted lines. The data management policy is declared in the "Availability of data and materials" section of this report.

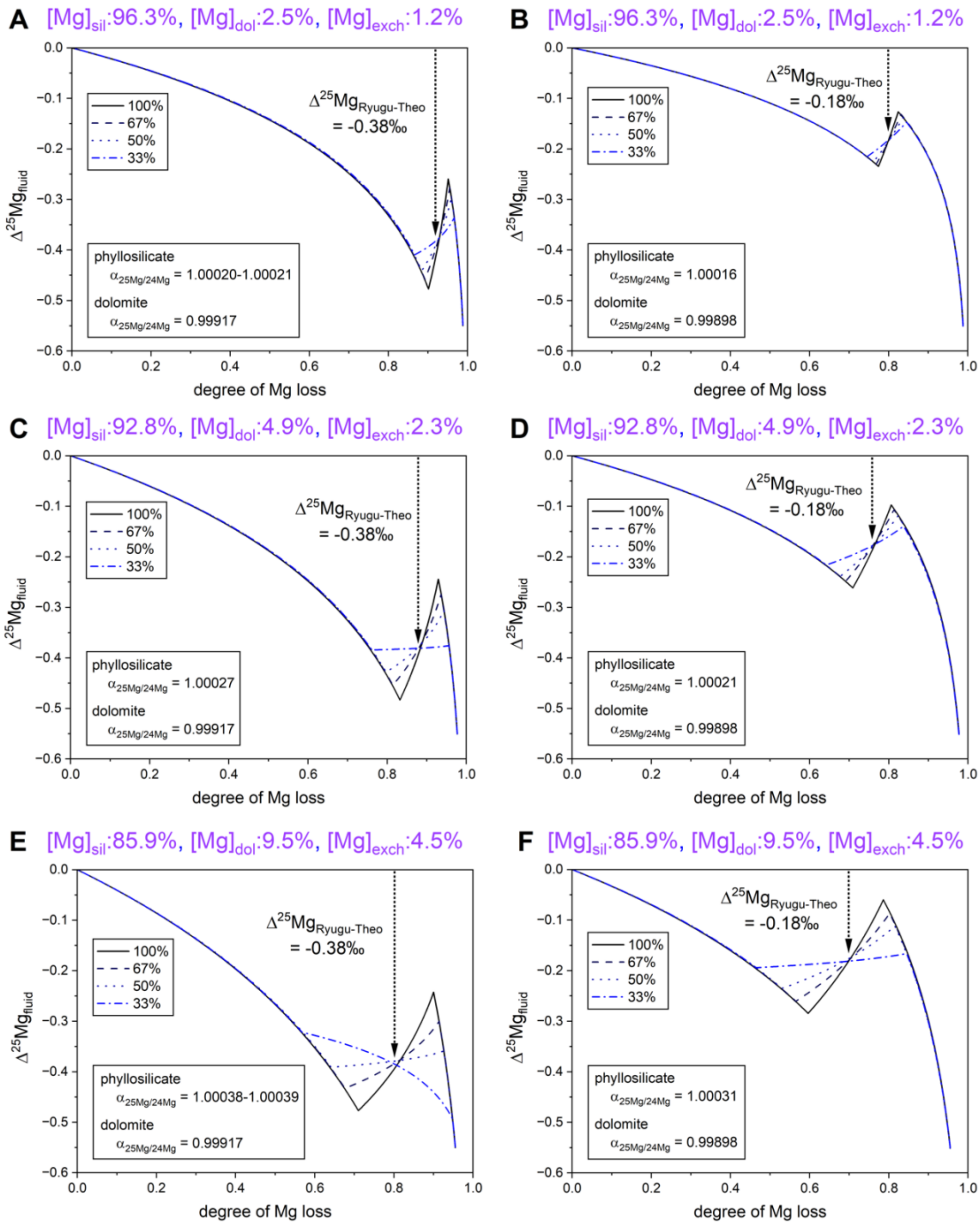

Supplementary Figure S6. Models of Mg partitioning calculated from changes in magnesium isotopic ratios. Mg partitioning ratios for phyllosilicate, dolomite, and exchangeable fractions are 96.3:2.5:1.2 for (A) and (B), 92.8:4.9:2.3 for (C) and (D), and 85.9:9.5:4.5 for (E) and (F). Magnesium isotopic fractionation factors for dolomite are cited in the calculations, with (A), (C), and (E) using values from laboratory precipitation experiments[11] and (B), (D), and (F) using values calculated from reaction transport

models for sediment and pore water[12] The difference between the line-types sets four conditions for the Mg-specific removal rates by precipitation of dolomite relative to phyllosilicate, ranging from the same condition for Mg precipitation rate for both phases (100%); one-third the precipitation rate for phyllosilicate (33%), i.e., the slower precipitation rate for dolomite. The isotopic fractionation coefficients for phyllosilicate were calculated to satisfy the isotopic ratio constraints obtained from the dolomite ($\Delta^{25}Mg_{Ryugu\text{-}Theo}$ = -0.38‰ or -0.18‰) and exchangeable fractions ($\Delta^{25}Mg_{fluid}$ at the endpoint is -0.55‰) and the model conditions described above.

Supplementary Tables

Supplementary Table S1. Percentages of clay mineral standards dissolved by the sequential extraction steps #10 and #9 conducted by Naraoka et al. (2023)[1] and by EDTA and CH_3COOH for carbonate leaching in this study. The percentage dissolution of the clay minerals is calculated by using the Al or Mg contents. The ratio of the solid to liquid phase of clay minerals is the same as the solid-to-liquid ratio of Naraoka et al. (2023)[1] (600 µL of reagent added for each ~15 mg of solids). "-" indicates no data available.

Reagent type	20% HCl		>99% HCOOH		6% CH_3COOH		EDTA-Na
Fraction number of Naraoka+2023	(#10)		(#9)				
Percentage dissolved	%	2SD	%	2SD	%	2SD	%
**Clay**							
Condition 1: 15 mg clay + 600 µL reagents							
JCSS-3501 Saponite	103.3%	2.3	10.6%	0.3	9.7%	0.1	<0.1
JCSS-3101b Montmorillonite	3.1%	0.1	0.5%	<0.1	1.6%	<0.1	<0.1
JCSS-2101 Pyrophyllite	0.6%	2.4	0.3%	<0.1	0.6%	<0.1	<0.1
JCSS-1301 Dickite	0.9%	<0.1	0.2%	<0.1	0.5%	<0.1	<0.1
JCSS-1101c Kaolinite	10.1%	0.2	1.6%	<0.1	2.6%	0.1	<0.1
Condition 2: 1 mg clay + 600 µL reagents							
JCSS-3501 Saponite	97.7%	2.5	-		-		-
JCSS-3101b Montmorillonite	6.6%	0.8	-		-		-
Condition 3: 75 mg clay + 600 µL reagents							
JCSS-3501 Saponite	80.6%	2.3	-		-		-
JCSS-3101b Montmorillonite	5.3%	0.3	-		-		-
**Carbonate**							
AIST JDo-1 Dolomite	-		103.3%	2.6	-		-

Supplementary Table S2. Molar ratios of the main component cations of the extraction solutions. Sodium was excluded from the calculation because the sample was contaminated with Na after the extraction of EDTA·2Na.

Sample	Mg mol%	Ca mol%	Na+K mol%
A0106, H_2O	0.373	0.039	0.589
C0107, H_2O	0.344	0.119	0.537
A0106, NH_4Cl	0.126	0.014	0.860
C0107, NH_4Cl	0.219	0.055	0.726

Sample	Mg mol%	Ca mol%	K mol%
A0106, EDTA	0.478	0.048	0.474
C0107, EDTA	0.551	0.159	0.289
A0106, CH_3COOH	0.735	0.247	0.018
C0107, CH_3COOH	0.758	0.234	0.007
A0106, HF+$HClO_4$	0.968	0.000	0.032
C0107, HF+$HClO_4$	0.994	0.000	0.006

Supplementary Table S3. Mg/Ca and Mg/Fe molar ratios of dolomite from Ryugu. Data compilation from Bazi et al. (2022)[46], Nakato et al. (2022)[47], Fujiya et al. (2023)[4], and Nakamura et al. (2023)[15]. "*n*" indicates the number of data acquisition points. "-" indicates no data available.

ID	n	Mg/Ca mol/mol	1SD	Mg/Fe mol/mol	1SD	Reference
A0058	49	0.978	0.041	12.67	1.15	Fujiya et al., 2023
A0106	-	0.917	-	16.61	-	Nakamura et al., 2023
C0002	52	0.978	0.042	10.83	1.57	Fujiya et al., 2023
C0033	-	0.911	-	11.05	-	Bazi et al., 2022
Q001	18	0.956	0.003	11.38	1.57	Nakato et al., 2022
**Average**		**0.948**		**12.51**		

Supplementary Table S4. Summary of Mg abundance and weighted average values of Mg isotopic ratios in the A0106 and C0107 cation exchange pools (H_2O and NH_4Cl fractions). The bold letters show the average value in each cation exchange pool and its offset from the bulk $\delta^{25}Mg$ value for C0108 given in Bizzarro et al. (2023)[16].

Sample	Mg fraction in the excheable pool	$\delta^{26}Mg$	2SD	$\delta^{25}Mg$	2SD
A0106					
H_2O	55.2%	-1.59	0.05	-0.85	0.02
NH_4Cl	44.8%	-1.19	0.09	-0.67	0.09
weighted Ave./Sum	100.0%	-1.41		-0.77	
C0107					
H_2O	62.1%	-1.35	0.04	-0.68	0.04
NH_4Cl	37.9%	-1.19	0.05	-0.61	0.04
weighted Ave./Sum	100.0%	-1.29		-0.65	
Average of A0106 and C0107		**-1.35**		**-0.71**	
Offset from Ryugu C0108 bulk (Bizzarro+)		n.d.		**-0.55**	

Supplementary Table S5. Mg isotope ratios and element ratios of Ryugu and meteorite samples reported by Yoshimura et al. (2023)[3] in the same format as Table 1. "-" indicates no data available.

Sample	δ^{26}Mg	2SD	δ^{25}Mg	2SD	δ^{26}Mg*	2SD	Mg/Ca (mol/mol)	Mg/Fe (mol/mol)	Mg/Al (mol/mol)	Mg/ Mg+Fe
A0106										
#9 HCOOH	-0.56	0.07	-0.287	0.05	0.004	0.003	9.71	2.33	67.70	70.0
#10 HCl	-0.26	0.09	-0.188	0.05	0.112	0.137	13.50	1.54	10.52	60.6
C0107										
#9 HCOOH	-0.65	0.07	-0.356	0.09	0.051	0.055	6.77	2.48	212.95	71.3
#10 HCl	-0.24	0.07	-0.144	0.02	0.045	0.053	20.25	1.46	9.84	59.4
Orgueil										
#5 H_2O	-1.42	0.08	-0.75	0.03	0.040	0.030	2.37	23545	724.46	100.0
#7-1 hot H_2O	-1.49	0.06	-0.77	0.01	0.021	0.005	2.74	40476	-	100.0
#7-2 hot HCl	-0.16	0.08	-0.09	0.08	0.013	0.014	29.05	1.11	10.46	52.6
#9 HCOOH	-1.32	0.09	-0.65	0.06	-0.049	0.082	4.14	0.27	59.60	21.2
#10 HCl	-0.07	0.07	-0.06	0.05	0.057	0.070	37.18	1.43	11.39	58.9
Other CCs										
Tarda, #9 HCOOH	-0.77	0.07	-0.38	0.03	-0.021	0.037	64.55	10.95	710.17	91.6
Tarda, #10 HCl	-0.19	0.06	-0.08	0.04	-0.034	0.046	16.40	1.49	10.84	59.8
Aguas Zarcas, #9 HCOOH	-0.66	0.07	-0.34	0.03	0.000	0.009	28.93	8.51	1096.34	89.5
Aguas Aguas, #10 HCl	-0.21	0.06	-0.08	0.04	-0.057	0.074	37.36	0.81	7.84	44.6
Jbilet Winselwan, #9 HCOOH	-0.56	0.07	-0.28	0.05	-0.014	0.026	2.01	6.07	516.63	85.9
Jbilet Winselwan, #10 HCl	-0.22	0.09	-0.10	0.05	-0.026	0.036	83.99	0.78	7.64	43.7
Serpentine										
#9 HCOOH	-0.42	0.07	-0.24	0.03	0.060	0.069	-	7.17	3.53	87.8
#10 HCl	-0.30	0.19	-0.19	0.02	0.078	0.094	-	0.99	8.98	49.7

Supplementary References

1. Naraoka, H. et al. 2023. Soluble organic molecules in samples of the carbonaceous asteroid (162173) Ryugu. Science 379, eabn9033.
2. Oba, Y. et al. 2023. Uracil in the carbonaceous asteroid (162173) Ryugu. Nature Communications, 14, Article number: 1292.
3. Yoshimura, T. et al. 2023. Chemical evolution of primordial salts and organic sulfur molecules in the asteroid 162173 Ryugu. Nature Communications 14, Article number: 5284.
4. Fujiya, W. et al. 2023. Carbonate record of temporal change in oxygen fugacity and gaseous species in asteroid Ryugu. Nature Geoscience 16, 675–682.
5. Dartois, E. et al. 2023. Chemical composition of carbonaceous asteroid Ryugu from synchrotron spectroscopy in the mid-to far-infrared of Hayabusa2-returned samples. Astronomy & Astrophysics 671, A2.
6. Moynier, F. et al. 2022. The Solar System calcium isotopic composition inferred from Ryugu samples. Geochemical Perspectives Letters 24, 1–6.
7. Ito, M. et al. 2022. A pristine record of outer Solar System materials from asteroid Ryugu's returned sample. Nature Astronomy 6, 1163–1171.
8. Yokoyama, T. et al. 2023. Samples returned from the asteroid Ryugu are similar to Ivuna-type carbonaceous meteorites. Science 379, eabn7850.
9. Schauble, E.A. 2011. First-principles estimates of equilibrium magnesium isotope fractionation in silicate, oxide, carbonate and hexaaquamagnesium(2+) crystals. Geochimica et Cosmochimica Acta 75, 844–869.
10. Rustad, J.R. et al. 2010. Isotopic fractionation of $Mg^{2+}_{(aq)}$, $Ca^{2+}_{(aq)}$, and $Fe^{2+}_{(aq)}$ with carbonate minerals. Geochimica et Cosmochimica Acta 74, 6301–6323.
11. Li, W., Beard, B.L., Li, C., Xu, H., Johnson, C.M. 2015. Experimental calibration of Mg isotope fractionation between dolomite and aqueous solution and its geological implications. Geochimica et Cosmochimica Acta 157, 164–181.
12. Fantle, M.S., Higgins, J. 2014. The effects of diagenesis and dolomitization on Ca and Mg isotopes in marine platform carbonates: implications for the geochemical cycles of Ca and Mg. Geochimica et Cosmochimica Acta 142, 458–481.
13. Higgins, J.A., Schrag, D.P. 2010. Constraining magnesium cycling in marine

sediments using magnesium isotopes. Geochimica et Cosmochimica Acta 74, 5039–5053.

14. Geske, A., Lokier, S., Dietzel, M., Richter, D. K., Buhl, D., Immenhauser, A. 2015. Magnesium isotope composition of sabkha porewater and related (sub-) recent stoichiometric dolomites, Abu Dhabi (UAE). Chemical Geology 393, 112–124.
15. Nakamura, T. et al. 2023. Formation and evolution of carbonaceous asteroid Ryugu: Direct evidence from returned samples. Science 379, eabn8671.
16. Bizzarro, M. et al. 2023. The magnesium isotope composition of samples returned from asteroid Ryugu. Astrophysical Journal Letters, 958, L25.
17. Takano, Y. et al. 2024. Primordial aqueous alterations recorded in water-soluble organic molecules from the carbonaceous asteroid (162173) Ryugu. Nature Communications. doi: 10.1038/s41467-024-49237-6.
18. Hashiguchi, M. et al. 2023. The spatial distribution of soluble organic matter and their relationship to minerals in the asteroid (162173) Ryugu. Earth, Planets and Space 75, Article number: 73.
19. Pogge von Strandmann, P.A.E. et al. 2012. Lithium, magnesium and silicon isotope behaviour accompanying weathering in a basaltic soil and pore water profile in Iceland. Earth and Planetary Science Letters 339–340, 11–23.
20. Tipper, E.T., Gaillardet, J., Louvat, P., Capmas, F., White, A.F. 2010. Mg isotope constraints on soil pore-fluid chemistry: evidence from Santa Cruz, California. Geochimica et Cosmochimica Acta 74, 3883–3896.
21. Wimpenny, J., Colla, C.A., Yin, Q.-Z., Rustad, J.R., Casey, W.H. 2014. Investigating the behaviour of Mg isotopes during the formation of clay minerals. Geochimica et Cosmochimica Acta 128, 178–194.
22. Wimpenny, J. et al. 2010. The behaviour of Li and Mg isotopes during primary phase dissolution and secondary mineral formation in basalt. Geochimica et Cosmochimica Acta 74, 5259–5279.
23. Fujiya, W., Sugiura, N., Hotta, H., Ichimura, K., Sano, Y. 2012. Evidence for the late formation of hydrous asteroids from young meteoritic carbonates. Nature Communications 3, Article number: 627.
24. Fujiya, W., Sugiura, N., Sano, Y., Hiyagon, H. 2013. Mn–Cr ages of dolomites in CI chondrites and the Tagish Lake ungrouped carbonaceous chondrite. Earth and

Planetary Science Letters 362, 130–142.

25. Hoppe, P., MacDougall, D., Lugmair, G.W. 2007. High spatial resolution ion microprobe measurements refine chronology of carbonate formation in Orgueil. Meteoritics & Planetary Science 42, 1309–1320.
26. McCain, K.A. et al. 2023. Early fluid activity on Ryugu inferred by isotopic analyses of carbonates and magnetite. Nature Astronomy 7, 309–317.
27. Nakamura, E. et al. 2022. On the origin and evolution of the asteroid Ryugu: A comprehensive geochemical perspective. Proceedings of the Japan Academy, Series B 98, 227–282.
28. Luu, T.-H., Hin, R.C., Coath, C.D., Elliott, T. 2019. Bulk chondrite variability in mass independent magnesium isotope compositions–Implications for initial solar system $^{26}Al/^{27}Al$ and the timing of terrestrial accretion. Earth and Planetary Science Letters 522, 166–175.
29. Kita, N.T. et al. 2013. ^{26}Al-^{26}Mg isotope systematics of the first solids in the early solar system. Meteoritics & Planetary Science 48, 1383–1400.
30. Young, E.D., Galy, A. 2004. The isotope geochemistry and cosmochemistry of magnesium. Reviews in Mineralogy and Geochemistry 55, 197–230.
31. Zhu, K. et al. 2023. Chondrite diversity revealed by chromium, calcium and magnesium isotopes. Geochimica et Cosmochimica Acta 342, 156–168.
32. Larsen, K.K. et al. 2011. Evidence for magnesium isotope heterogeneity in the solar protoplanetary disk. The Astrophysical Journal Letters 735, L37.
33. van Kooten, E.M. et al. 2016. Isotopic evidence for primordial molecular cloud material in metal-rich carbonaceous chondrites. Proceedings of the National Academy of Sciences 113, 2011–2016.
34. van Kooten, E., Cavalcante, L., Wielandt, D., Bizzarro, M. 2020. The role of Bells in the continuous accretion between the CM and CR chondrite reservoirs. Meteoritics & Planetary Science 55, 575–590.
35. Essington, M.E. 2003. Soil and Water Chemistry: An Integrative Approach. CRC press, pp. 1–534.
36. Kleber, M., Sollins, P., Sutton, R. 2007. A conceptual model of organo-mineral interactions in soils: self-assembly of organic molecular fragments into zonal structures on mineral surfaces. Biogeochemistry 85, 9–24.

37. Sreedhara, A., Cowan, J.A. 2002. Structural and catalytic roles for divalent magnesium in nucleic acid biochemistry. Biometals 15, 211–223.
38. Parker, E. T. et al. 2023. Extraterrestrial Amino Acids and Amines Identified in Asteroid Ryugu Samples Returned by the Hayabusa2 Mission. Geochimica et Cosmochimica Acta 347, 42–57.
39. Tachibana, S. et al. 2022. Pebbles and sand on asteroid (162173) Ryugu: In situ observation and particles returned to Earth. Science, 375, 1011–1016.
40. Yada, T. et al. 2022. Preliminary analysis of the Hayabusa2 samples returned from C-type asteroid Ryugu. Nature Astronomy 6, 214–220.
41. Sakamoto, K. et al. 2022. Environmental assessment in the prelaunch phase of Hayabusa2 for safety declaration of returned samples from the asteroid (162173) Ryugu: Background monitoring and risk management during development of the sampler system. Earth, Planets and Space 74, Article number: 90.
42. Yabuta, H. et al. 2023. Macromolecular organic matter in samples of the asteroid (162173) Ryugu. Science 379, eabn9057.
43. Urashima, S.-h., Nishioka, T., Yui, H. 2022. Micro-Raman spectroscopic analysis on natural carbonates: linear relations found via biaxial plotting of peak frequencies for cation substituted species. Analytical Sciences 38, 921–929.
44. Merlini, M., Hanfland, M., Gemmi, M. 2015. The $MnCO_3$-II high-pressure polymorph of rhodocrosite. American Mineralogist 100, 2625–2629.
45. Yada, T. et al. 2023. A curation for uncontaminated Hayabusa2-returned samples in Extraterrestrial Curation Center of JAXA: From the beginning to nowadays. Earth Planets Space, 75, Article number: 170.
46. Bazi, B. et al. 2022. Trace-element analysis of mineral grains in Ryugu rock fragment sections by synchrotron-based confocal X-ray fluorescence. Earth, Planets and Space 74, Article number: 161.
47. Nakato, A. et al. 2022. Ryugu particles found outside the Hayabusa2 sample container. Geochemical Journal 56, 197–222.
